# Effect of Individual, Simultaneous and Sequential Inoculation of *Pseudomonas fluorescens* and *Meloidogyne incognita* on Growth, Biochemical, Enzymatic and Nonenzymatic Antioxidants of Tomato (*Solanum lycopersicum* L.)

**DOI:** 10.3390/plants10061145

**Published:** 2021-06-04

**Authors:** Ahmed Noureldeen, Mohd Asif, Taruba Ansari, Faryad Khan, Mohammad Shariq, Faheem Ahmad, Manar Fawzi Bani Mfarrej, Amir Khan, Moh Tariq, Mansoor Ahmad Siddiqui, Amal Al-Barty, Hadeer Darwish

**Affiliations:** 1 Department of Biology, College of Sciences, Taif University, Taif 21944, Saudi Arabia; a.noureldeen@tu.edu.sa (A.N.); aalbarty@tu.edu.sa (A.A.-B.); 2 Plant Pathology and Nematology, Department of Botany, Aligarh Muslim University, Aligarh 202002, India; tarubaansari11@gmail.com (T.A.); khanfaryadamu@gmail.com (F.K.); ansarishariq.amu@gmail.com (M.S.); alig.faheem@gmail.com (F.A.); amirsz2504@gmail.com (A.K.); mansoor_bot@yahoo.co.in (M.A.S.); 3 Pharmacopoeia Commission for Indian Medicine and Homeopathy, Ghaziabad 201002, India; 4 Department of Life and Environmental Sciences, College of Natural and Health Sciences, Zayed University, Abu Dhabi 144534, United Arab Emirates; manar_mfarrej@yahoo.com; 5 Department of Botany, Lords University, Alwar 301028, India; aziztariq14@gmail.com; 6 Department of Biotechnology, College of Sciences, Taif University, Taif 21944, Saudi Arabia; hadeer@tu.edu

**Keywords:** *Solanum lycopersicum*, tomato, antioxidant enzymes, inoculation, *Meloidogyne incognita*, *Pseudomonas fluorescens*, phenol content, modulation

## Abstract

This study was conducted on tomato (*Solanum lycopersicum* cv. K-21) to investigate the bioprotective nature of *Pseudomonas fluorescens* and its interactive effects with *Meloidogyne incognita* in terms of growth biomarkers, changes in biochemical attributes and modulation in antioxidant enzymes of the tomato plant. In this study, we grew tomato plants with *M. incognita* and *P. fluorescens* in separate pots, simultaneously and sequentially (15 days prior or post) after 15 days of seed sowing. The sequential inoculation of Mi15→Pf maximally increased the root-knot index and decreased the nematode population. It was also noted that inoculation suppressed the plant growth biomarkers in comparison to control. However, maximum suppression in nematode reproduction and increment in growth and physiological attributes were observed when *P. fluorescens* was applied 15 days prior to the nematode (Pf15→Mi) as compared to control. All the treatments showed an increase in antioxidant enzymes. Expression of phenol content and defensive enzymes such as peroxidase (POX) and superoxide dismutase (SOD) increased, in contrast to a significant reduction in malondialdehyde (MDA) and hydrogen peroxide (H_2_O_2_) contents when compared with the untreated inoculated plants. However, the highest levels of POX and SOD, and a lowest of phenol, MDA and H_2_O_2_ were displayed in the treatment Pf15→Mi, followed by Mi+Pf and Mi15→Pf.

## 1. Introduction

Phytonematodes are considered among the most devastating agricultural pests, causing a disastrous deficit of 12–15% of world annual crop yield [1]. They infest and attack a wide range of agriculture crops leading to yield loss [2]. Nematode infestations often lead to root dysfunction, which eventually reduces the utilization efficiency of water and nutrients in crops [3]. India ranks second, after China, in tomato production with an annual production of 18.7 million tons, occupying an area of 882,030 hectares [4].

The tomato agricultural industry faces several major problems which lead to low crop yield. This low yield in tomato crops is caused by various abiotic and biotic factors including nematodes, bacteria, fungi and viruses. The root-knot nematode *Meloidogyne incognita* is a destructive pest that reduces tomato yields by 25.0% and up to 49.0% [5]. Although using nematicides can effectively deal with nematode infestations, the harmful impact they have on the environment, and pollution, have led to rising concerns and the search for safe and environmentally friendly alternatives for the management of phytonematodes. One environmentally sustainable option for managing the root-knot nematode lies in the exploration of biological pest control [6].

Biocontrol technologies offer alternative approaches to chemical or cultural control of nematodes and improving plant growth in nematode-infected plants. Plant growth-promoting rhizobacteria (PGPR) have been shown to be reliable biologically regulating microbes for the phytonematode populations [7]. In nature, endophytic bacteria dwell in plant root tissues alongside nematodes through their life cycles, controlling their population. This makes endophytic organisms ideal candidates for use as biocontrol agents as they are often endemic to plant roots and are naturally utilized in this manner in the wild. They suppress the nematode populations using a wide range of chemicals with several mechanisms such as creating toxins, antimicrobials and chemicals, or affecting nematode-plant-host identification [8].

*Pseudomonas fluorescens* includes a class of normal, nonpathogenic microbes that are prevalent in soil, water and plants. Numerous species of *Pseudomonas* are known to be plant growth-promoting rhizobacteria (PGPR) which also reduce the population of deleterious microorganisms and enhance N_2_ fixation. Nitrogen fixation is a rare feature in the genus *Pseudomonas*. This is due to several reasons. First, *Pseudomonas* could be a favorable background for the expression of a heterologous nitrogenase enzyme. Second, the horizontal transfer of nitrogen fixation in *Pseudomonas* strains has occurred and all the genes required for the expression of nitrogenase could be efficiently packaged within the nitrogen fixation islands of the *Pseudomonas* strains. This is supported by indirect evidence such as the global transcriptional analysis of nitrogen [9]. They also solubilize nutrients such as phosphorus, while boosting mycorrhizal behavior, managing ethylene production in roots and increasing the production of phytohormones linked to induced systemic resistance in plants [10].

Model organisms such as *P. fluorescens* have shed a light on the molecular phenomenon of plant defense induced by PGPR. *Pseudomonas* spp. stifle disease by expanding the movement of protective enzymes and proteins, that lead to a reduction in oxidative stress [11]. The oxidative burst, which results in the release of ROS, is a hallmark of plant defense response to pathogen infection and it acts as an essential signal for subsequent defenses [12]. Plants defend themselves by the synthesis or induction of different enzymatic or nonenzymatic antioxidants from this oxidative stress [13]. The most common phenomenon for detoxifying ROS synthesized amid stress reactions is the induction of ROS-defending enzymes such as superoxide dismutase (SOD), peroxidases (POXs) and catalase (CAT) [14]. Various studies reported that phenolic compounds like polyphenol oxidase (PPO) serve as antioxidants for the management of plant disease [15,16]. In plants, nitric oxide (NO) is a gaseous secondary messenger that is critical for proper cell signaling and plant survival when exposed to stress and is involved in important physiological processes such as defense processes and plant–pathogen interactions. Moreover, the hypothesis that molybdoenzyme nitrate reductase (NR) is the main enzyme responsible for NO production in most plants has been investigated [17,18].

Therefore, the present investigation tests the effect of *Pseudomonas fluorescens* on tomato, its performance in the protection from the root-knot nematode, *M. incognita*, and the modest biochemical alteration that occurs in the host during infection.

## 2. Results

### 2.1. Impacts of Inoculation of Meloidogyne incognita and Pseudomonas fluorescens on Growth Parameters of Tomato

In this study, experiments were carried out to understand the role of *P. fluorescens* against *M. incognita* and to measure the alteration in photosynthetic pigments, phenols and antioxidant enzymes. This study showed that these interactions modified various growth and physiological parameters of the plants. Among the treatments there was a significant difference (*p* ≤ 0.05) in plant growth characters and nematode infestation level.

**Figure 1:**
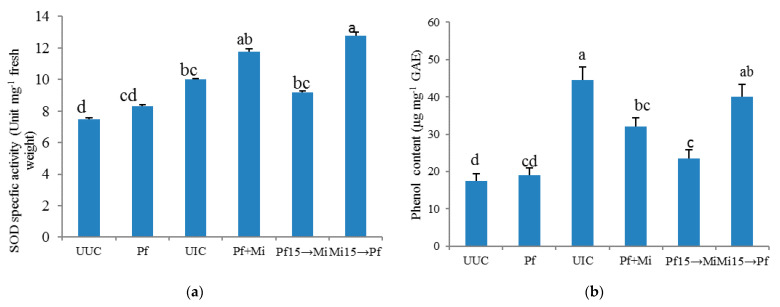

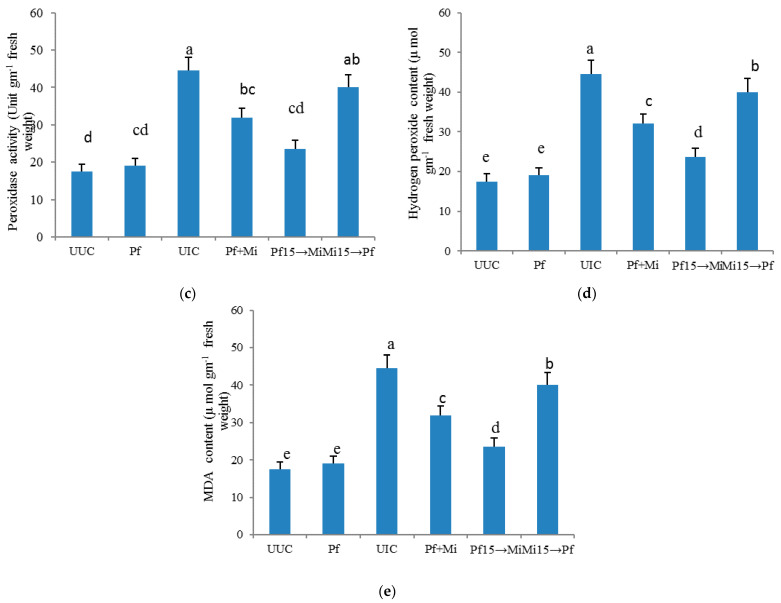
Effect of inoculation of *P. fluorescens* and *M. incognita* on physiological attributes in tomato. (**a**) SOD specific activity (**b**) phenol content (**c**) peroxidase activity (**d**) H_2_O_2_ content (**e**) MDA content. → Indicate the sequence of inoculation of *M. incognita* (Mi)/*P. fluorescens* (Pf) 15 days prior. + Indicates simultaneous inoculation. UIC: Untreated inoculated Control. UUC: Untreated Uninoculated Control. Statistical analysis was done using SPSS17.00. Means in each column followed by the same letter are not significantly different according to Duncan’s Multiple Range Test (DMRT) at *p* ≤ 0.05.

The exploration of the biocontrol agent significantly increased growth biomarkers in terms of weight and height of plants and roots (Table 1). Growth biomarkers were affected by the infection of *M. incognita* (Mi) either alone or combined with *P. fluorescens* (Pf). Application of *P. fluorescens* alone (Pf) showed the most significant (*p* ≤ 0.05) improvement in plant growth matters. However, the greatest reduction in plant growth biomarker was displayed by *M. incognita* (Mi) inoculation alone. It was followed by sequential inoculation of *M. incognita* preceded by 15 days to *P. fluorescens* (Mi15→Pf), and *P. fluorescens* inoculation preceded by 15 days to *M. incognita* (Pf15→Mi). Moreover, simultaneous inoculation of (Mi+Pf) suppressed the growth matters up to a limited extent. Nematode suppressed plant growth matters in the order of (Mi) > (Mi15→Pf) > (Mi+Pf) > (Pf15→Mi) > (Pf) (Table 1 and Figure 1).

### 2.2. Impacts of Inoculation of Meloidogyne incognita and Pseudomonas fluorescens on Yield and Biochemical Parameters of Tomato

All the treatments were significantly effective (*p* ≤ 0.05) in enhancing the biochemical parameters of the tomato plants. Individual application of *P. fluorescens* (Pf) significantly (*p* ≤ 0.05) increased biochemical parameters in terms of chlorophyll, carotenoid and nitrate reductase content as compared to the untreated inoculated control plants. However, the individual application of *M. incognita* (Mi) resulted in the highest significant (*p* ≤ 0.05) reduction in biochemical parameters. Concurrent and sequential use of nematode and bacterium significantly (*p* ≤ 0.05) enhanced the biochemical parameters. Subsequent application of *P. fluorescens* to plants 15 days before *M. incognita* (Pf15→Mi) was significantly (*p* ≤ 0.05) effective in the improvement of physiological parameters as compared to *M. incognita* preceded by *P. fluorescens* by 15 days (Mi15→Pf). Still, in the application of the *P. fluorescens* and *M. incognita* combination (Pf+Mi), a limited but significant (*p* ≤ 0.05) reduction was observed (Table 2).

In another study, the individual application of *M. incognita* showed the most significant improvement in the pollen fertility and yield of the plant. The sequential application of *P. fluorescens* and *M. incognita* also showed considerable enhancement. Still, the use of *P. fluorescens* 15 days before *M. incognita* (Pf15→Mi), showed the most considerable increase in contrast to *M. incognita* 15 days before *P. fluorescens* (Mi15→Pf) which displayed the least significant improvement compared to the untreated inoculated control. Concomitant application (Pf+Mi) also caused a substantial increase in the pollen fertility and yield, but the results in yield and pollen fertility were not satisfactory (Table 2).

It was observed that the phenol content, MDA content, hydrogen peroxide content (H_2_O_2_) and antioxidant defense enzymes (POX and SOD) were higher in all treated plants. Maximum phenol content, POX and SOD activities were recorded in Mi15→Pf plants followed Mi15+Pf and UIC. However, the maximum H_2_O_2_ and MDA were recorded in control plants. The plants inoculated with *M. incognita* (Mi) exhibited significant phenol content. Similar significant (*p* < −0.05) increases were observed in SOD and peroxidase activity. However, the highest increment observed was in H_2_O_2_ and MDA production. Subject to the application of plants with *P. fluorescens*, almost identical and nonsignificant phenol content was detected. In the same condition, no significant increase was found for the SOD, peroxidase, H_2_O_2_ and MDA. In the case of the other application, when *M. incognita* was applied 15 days previously to *P. fluorescens* (Mi15→Pf), the increase in content, as observed for the H_2_O_2_ and MDA, compared to the untreated uninoculated control plants. However, the highest and statistically most significant (*p* ≤ 0.05) phenol content, SOD and POX activities were observed under similar conditions as compared to the control. Moreover, in reciprocal cases (Pf15→Mi) phenol, H_2_O_2_ and MDA were significantly increased in comparison to the untreated inoculated control. Furthermore, no significant increase was detected for SOD and peroxidase under the same conditions. In the case of simultaneous inoculation of Mi+ Pf, significant (*p* ≤ 0.05) increases in the content of phenol, SOD, H_2_O_2_ and MDA were observed. However, no significant increase in peroxide activity was detected under similar conditions (Figure 1).

### 2.3. Impacts of Inoculation of Meloidogyne incognita and Pseudomonas fluorescens on Nematode Multiplication of Tomato

The nematode induced parameters (eggmasses/root, eggs/eggmass, nematode population and root-knot index) on host plant were reduced significantly in the presence of *M. incognita* (Pf) as compared to the untreated control plant. Results depicted that nematode induced parameters were reduced (*p* ≤ 0.05) in plants when using inoculation made with Mi+ Pf in comparison to the untreated inoculated plants (Table 3), but the suppression of nematode infestation was not to the same extent. However, the greatest nematode induced parameters were observed when Mi was applied 15 days before Pf (Mi15→Pf). When Pf was administered 15 days before Mi (Pf15→Mi), no increases in the nematode induced parameters were observed against *M. incognita* alone (Mi) (Table 3)

## 3. Discussion

In this study, the interaction of *Pseudomonas fluorescens* with a tomato plant and the function of *M. incognita* in growth-promoting activity, and its nature as a defense activator against the root-knot *M. incognita,* were studied. Modifications in growth, biochemical and antioxidant enzymes of the plants, due to the interaction in this plant, were observed. Growth parameters and yield of the treated plants increased compared to the untreated inoculated plants. Similar observations were reported in tomato [19] infected with *Meloidogyne* spp. The results of this study showed that *M. incognita*-inoculated plants had increased growth biomarkers such as plant length, fresh weight and dry weight in addition to the yield. This increase in biomass might be due to the production of phytohormones, which may indirectly assist in the enhancement of root growth, allowing for a higher uptake of water and nutrients, which increase plant growth and yield [20]. Wei et al. [21] revealed that some species of Pseudomonas produce siderophore which supports plant growth.

The application of *M. incognita* significantly improved the biochemical parameters compared to the untreated inoculated control plants. These results agree with previous report of [22]. The same observation for the enhancement of Chlorophyll a, b and carotenoids levels in Pseudomonas-treated plants were reported by Rani [23]. Exaggeration of nitrate reductase activity indicates an increase in the concentration of NH_3_, which is used by a-ketoglutarate to form glutamic acid, and which might be used as a sink for the synthesis of other amino acids [24].

The exploration of tomato plant roots by *Pseudomonas fluorescens* significantly increased the tolerance and reduced the susceptibility of it to *M. incognita*. Applications of *P. fluorescens* 15 days before *M. incognita* (Pf15→Mi) significantly exaggerated the growth biomarkers, and plants yield compared to the *M. incognita* inoculation 15 days before the *P. fluorescens*. The exaggeration in growth biomarkers and yield was greater when the plants were treated with bioagents, as they had sufficient time to colonize the root tissue. After colonisation, toxic secretion of the applied bioagent produced an antagonistic effect on nematodes and increased the availability of atmospheric nitrogen to the plants [25]. The findings of the study are in support of previous research work by Parveen et al. [26]. The initial application of fluorescent pseudomonads before invasion protects the crop from the pathogens by strengthening the cell wall structure and triggering biochemical and physiological changes in the plant system. Moreover, *M. incognita* inoculation prior to *P. fluorescens*, (Mi15→Pf) caused a reduction in growth biomarkers, as the nematode had adequate time to multiply and to infect the plants root system. This led to mandatory changes and resulted in limiting the effect of bioagents inoculated later [27]. The results of this study are like the previous study of Ganaie and Khan [28].

Application of *M. incognita* 15 days before *M. incognita* (Pf15→Mi) showed significant improvement in chlorophyll, carotenoid and nitrate reductase levels compared to the decline of *M. incognita* 15 days preceded by *P. fluorescens*. The findings of this study revealed that the biochemical pigments were highly sensitive to the alteration in host physiology induced by *M. incognita*. Biotic stress caused by the nematode in plants resulted in water stress due to root damage and development of galls [29]. This biotic stress disturbs the plant physiology and has been considered the reason behind the reduction of photosynthetic activity in nematode-infected plants [30]. The plant showed a decrease in chlorophyll content and photochemical limitations [31]. Most importantly, the failure to degrade the chlorophyll may cause an accumulation of reactive oxygen species (ROS) that can damage cellular organelles [12]. The activity of nitrate reductase (NRA) in plants provides a sensible estimate of N status and is often correlated with growth and yield of crops [32]. ROS interact with nitric oxide (NO) and mediate the responses to different environmental situations, even promoting the systemic adaptation of plants to stress situations [33]. Two main pathways, reductive and oxidative, appear to explain NO synthesis in plants. One is based on the reduction of nitrite, and the other involves the oxidation of aminated molecules such as the amino acid arginine [34].

Paramount decrease in nematode-induced parameters such as eggs, egg masses, the nematode population and the root-knot index, were observed in the sequential inoculation of *P. fluorescens* 15 days before root-knot nematodes (Pf15→Mi). Inoculation of *P. fluorescens* in the plants reduced the nematode population, which might have been due to colonization of bacteria around the roots. This colonization created unfavourable conditions for the nematodes to enter the roots. Additionally, this could be possible due to the toxic secretion of bacteria, which had an antagonistic effect on nematodes and made the plants less susceptible to nematode attack. Similar findings were confirmed by Burelle and Samas [35] and Parveen et al. [26].

A previous investigation carried out by Siddiqui and Shaukat [36] showed the production of metabolites by rhizospheric bacteria. These studies reported that lysis of nematode eggs and reduction in the attraction or degradation of specific root exudates led to changes in nematode behaviour. *M. incognita* 15 days preceded by *P. fluorescens* (Mi15→Pf) treatment caused the least significant suppression, in terms of eggs, eggmasses, nematode population and root-knot index. These higher levels of root-knot indices demonstrated that the infection by a pathogen such as the nematode prior to *P. fluorescens* would support the synergistic interaction consequently, resulting in more significant plant damage (1 + 1 > 2). The results of the current study were consistent with the findings of Safiuddin et al. [37] who reported a synergistic effect on the rate of nematode reproduction between the root-knot nematode and *Rhizoctonia solani*.

Concomitant application of *P. fluorescens* and *M. incognita* (Pf+Mi) showed antagonistic effects in plants that favored the improvement in growth characters as compared to the untreated inoculated control plants. This might be justified due to the enhancement of t mineralization processes, specifically nitrogen uptake and assimilation [38]. Nitrogen is a critical component as it affects pest infestation in plants and the host selection process. Pests respond to total host nitrogen level in addition to nitrogen form and its availability [39]. According to Sarathchandra et al. [40], populations of *Meloidogyne* spp. were reduced in soils fertilized with nitrogen compared to unfertilized soils. *Pseudomonas* spp. were associated with the production of siderophores, exopolysaccharides, ammonia and hydrogen cyanide (HCN), which is known as prussic acid and released as a plant defense mechanism [39,41].

The current study showed a noticeable (*p* ≤ 0.05) reduction in nematode induced parameters in plant roots compared to untreated inoculated plants. This might be due to a reduction in nematode population by the presence of rhizobacteria, mainly by triggering ISR, competing for the essential nutrients and regulating nematode behaviour [42], or directly through an antagonizing effect by the production of toxins, enzymes and other metabolic products [43].

On the other hand, individual inoculation of *M. incognita* caused the most significant reduction in plant growth biomarkers and plants yield compared to uninoculated controls. Besides this, decreases in chlorophyll, carotenoid and nitrate reductase activity were observed in infected tomato plants in the current study (Table 2). The detrimental role of the phytoparasitic nematodes on the plants caused a reduction in plant growth. The retarded growth of infected plants might be explained by the scarcity of food, as plant organs did not receive metabolites in required quantities [44]. Disruption of vascular tissues due to the infection of nematodes reduced the transportation of water and nutrients to the foliar systems, which in turn reduced the photosynthetic rates in the plants. Nematodes affected root uptake rate and mineral translocation and, therefore, changed plant element concentrations that directly or indirectly affect the host physiology, plant yield and crop nutrition [45].

Phenol content, H_2_O_2_, MDA and antioxidant enzyme activities were studied in the tomato plants shoots inoculated with *P. fluorescens* and *M. incognita*. The findings suggest that plant colonized with *M. incognita* alone stimulated phenol production over control. An increase in phenol content was reported to be a contributing factor in developing resistance in host plants during nematode infections [46]. It has been reported that the increase in the phenolic concentration can be attributed to the high synthesis of H_2_O_2_ due to enhanced respiration or by stimulation of the hexose-monophosphate pathway and acetate pathway in addition to the discharge of phenols through hydrolytic enzymes in elicited plant cells [47]. Under stress conditions, phenolic metabolism participates in obstructing the growth of the pathogen and results in increasing host cell endurance [48]. The increases in phenol concentration caused by pathogen invasion activates mRNA transcription, which in turn codes for phenylalanine ammonia-lyase (PAL) resulting in an increase of the amounts of PAL in the plant, which brings about the synthesis of phenolic compounds [49].

The same path of induction of SOD and peroxidase activities, as well as H_2_O_2_ and MDA contents, in plants inoculated with *M. incognita* was noted, as in the case of phenol content. It is suggested that greater generation of ROS in tomato plants, especially H_2_O_2_ as a result of pathogen infection, appears to be a critical step of disease-resistance mechanisms. Induced lipid peroxidation might be one of the mechanisms that lead to cell death [50]. Similarly, it has been reported that induction of H_2_O_2_ by oxidative stress plays a critical role in the orchestration of localized hypersensitive responses during plant disease resistance expression [51]. The excess H_2_O_2_ produced during plant-pathogen interactions, suggests its direct role as an antimicrobial agent and the cause of localized membrane damage at the infection site [11]. An increase in MDA levels in plants as a result of infection with RKNs is considered normal, due to stress conditions. Therefore, in the present study, the increase displayed in H_2_O_2_ production in nematode-inoculated plants compared to *P. fluorescens*-treated and untreated uninoculated plants, serves as the defense mechanism against the root-knot nematode infestation.

In a study conducted by Kuzniak and Skłodowska [52], nematode infection caused a marked increase in antioxidant enzymes (POX and SOD) activity. Increased activity of antioxidant enzymes minimized the chances of an oxidative burst, and consequently *P. fluorescens* might be protected from the oxidative defense system during colonization. It has been reported that enhancement in POX activity in plants is critical in strengthening the plant cell walls at the border of infection, which is considered an important component of an active defense response in the nematode-invaded tissue [53].

The activities of antioxidant enzymes, lipid peroxidation and H_2_O_2_ showed variability in response to sequential and concomitant inoculation of *P. fluorescens* and *M. incognita*. In the current study, the augmentation in phenol content, POX and SOD were detected after sequential and concomitant inoculation of *P. fluorescens* and *M. incognita*. However, suppression in MDA content was observed in all the three conditions of sequential and concomitant inoculation. The study demonstrated that significant exaggeration was reported in phenol content and other plant defense enzyme (POX and SOD) production compared to the nematode-infected plants. The higher production of defensive compounds might favor resistance to nematodes in tomato plants. In plant responses to pathogen infection, the O_2_**^.^**^-^ generated is usually dismutated rapidly via SOD to H_2_O_2_. During this process, the plant cell wall becomes more resistant to pathogen penetration and enzymatic degradation. Similar findings were reported by Anita and Samiyappan [54] in bacterized rice plants, where induction of SOD activity was detected against the rice root-knot nematode, *Meloidogyne graminicola*, which resulted in the suppression of nematode development. A similar observation of POX, SOD and phenolics production was recorded in rice roots inoculated with *M. incognita* and the rice root-knot nematode *M. graminicola* [55]. In addition, Shabaev et al. [56] observed that *M. incognita* releases antimicrobial factors including lytic enzymes, which results in the accumulation of phenolics by secretion of indole acetic acid that induces phenol metabolism in plants.

The current study also showed that oxidative damage and ROS accumulation were due to the stress caused by the reproduction of the root-knot nematode depicted in the form of MDA content of the plant. Enhanced peroxidation was detected under the nematode stress condition, but inoculation of *P. fluorescens* decreased the peroxidation, thereby protecting the cell from membrane destruction. This finding confirms that a significant reduction in MDA content was observed after the sequential and concurrent inoculation of *M. incognita* compared to the untreated inoculated control plants. These results confirmed the investigation of Labudda et al. [57] on the effects on barley caused by two pests, cereal cyst nematode, *Heterodera filipjevi* and wheat curl mite, *Aceria tosichella* infesting separately or at once, and verified the hypothesis about the involvement of redox metabolism and photosynthesis in barley defence responses.

Furthermore, the application of *P. fluorescens* could alleviate nematode stress and may repair membrane destruction. The reduction in MDA content in *P. fluorescens*-inoculated plants under the nematode stress conditions compared to non *Pseudomonas*-inoculated plants, could be explained by the enhanced POX, SOD enzyme activities, and phenol contents. The effect can be evident with the results obtained by Sundararaju and Suba [58].

The findings of this study indicate that *M. incognita* and *P. fluorescens* showed an antagonistic relationship. The presence of *P. fluorescens* decreased the *M. incognita* infection in tomato plants, which might be due to the increased levels of antioxidants, phenol content, H_2_O_2_ and photosynthetic pigments levels, as well as by suppressing the oxidative damage done by the reduction of MDA and H_2_O_2_ content. The application of *P. fluorescens* avoids the use of synthetic pesticides through exaggeration of plant growth biomarkers, yield and induction of systemic resistance. The study also shows that *P. fluorescens*-inoculated tomato seems to be more resistant to biotic stress, which leads to it not negatively affecting the plant biomass and yield. In conclusion, the formulation of a microbial elicitor involving *P. fluorescens* is an environmentally safe and sound approach used in sustainable nematode management programs.

## 4. Materials and Methods

Around 100 seeds of tomato cv. K-21 were placed in a 0.01% mercuric chloride solution for two minutes followed by washing of the seeds four times with refined water (DW). The experiments were performed in the Botany Divison of Aligarh Muslim University, Aligarh, under greenhouse conditions.

### 4.1. Nematode Culture

A standard culture of *Meloidogyne incognita* was started with a well-identified fresh eggmass, initially extracted from an eggplant farm close to Punjipur town, Aligarh (U.P). Species morphology was identified based on perineal pattern preparations [59]. The extracted eggmass was followed by the mass-culturing on cv. BR-112 of brinjal in a nursery of the Division of Botany, A.M.U., Aligarh. The pure culture of *M. incognita* was maintained at 25 ± 1 °C in the greenhouse.

### 4.2. Culture of Bacterium Inoculum

Genuine *Pseudomonas fluorescens* culture (ITCC No. BE0004) was procured from IARI, New Delhi. The method of Vidhyasekaran and Muthamilan [60] was used for the preparation of a formulation by using a mixture of 10 g carboxymethyl cellulose and 1 kg talc. Calcium carbonate (15 g) was added to maintain the pH at 7.0, and the mixture was sterilized for 30 min for two successive days. The culture of *M. incognita* was developed on liquid King’s B medium (KBM) in an Infors AG shaker at 150 rpm for 48 hrs at 25 ± 2 °C. After that, one kg talc was mixed with 400 mL bacterial suspension containing 2 × 10^6^ CFU/mL under sterile conditions, and moisture was reduced to below 20% by drying.

### 4.3. Role of M. incognita in Bio-Protection

To study the bioprotective nature of *M. incognita*, plants were initially grown for two weeks before the inoculation. As the root was stabilized in the soil, the following set of treatments were prepared. (1) tomato plants explored on day one with *P. fluorescens* alone and cultivated for two months; (2) tomato plants initially explored on day one with *P. fluorescens*, and on day 15 with *M. incognita* and cultivated for two months; (3) tomato plants initially inoculated on day one with *M. incognita* and on day 15 explored with *P. fluorescens* and cultivated for two months; (4) tomato plants inoculated simultaneously on day one with both *P. fluorescens* and *M. incognita* and cultivated for two months; (5) tomato plants inoculated with *M. incognita* alone on day one and grown for two months; (6) tomato plants grown for two months without any treatment (control). In relation to variation in time, sequential and concomitant inoculation of nematode and bacterium were chosen to evaluate the protective act of the bacterium by restraining the nematode infestation and determining whether there was any effect on the biomass, or any alteration in the morphology, physiology and antioxidant enzyme activity of the plant. The growth-promoting effect of the bacterium was examined by analyzing the growth attributes irrespective of the inoculated control. The experiment was performed with five replicates each in a fully randomized design. The experiment was repeated once. The plants inoculated with *M. incognita* and *M. incognita* in an individual, sequential and simultaneous order were arranged according to the following experimental design:

Pf- Inoculated with *Pseudomonas fluorescens* at 3.0 g/pot alone.

Pf+ Mi—Inoculated with *P. fluorescens* (3.0 g) + *M. incognita* (1500 J_2_) simultaneously

Mi→Pf- Inoculated with *M. incognita* (1500 J_2_) 15 days prior to P. fluorescens (3.0 g)

Pf→Mi—Inoculated with *P. fluorescens* (3.0 g) 15 days prior to *M. incognita* (1500 J_2_)

Mi—Inoculated with M. incognita (1500 J_2_) alone

Untreated Uninoculated Control (UUC)

### 4.4. Pot Experiment

The cultivar K-21 grown with the aim of productivity by local farmers at the commercial level, has four leaves and was selected for the experiments. The pots were filled with 1 kg of autoclaved sterile sandy loamy soil in the ratio 3:1 (sand loam: farmyard manure). A nematode suspension in sterile distilled water was inoculated with a pipette to seedlings at the rate of 1500 s stage juveniles (J_2_s) of *M. incognita* by making 4–5 pits into the plant’s rhizosphere without damaging the root system. The plants were watered regularly to maintain plant health. At the young stage, pollen fertility (%) was estimated to determine productivity. The phenol content, MDA content, hydrogen peroxide content (H_2_O_2_), and antioxidant defense enzymes (POX and SOD) were measured in 20-day old nematode infected *Solanum lycopersicum* plants. The impact on different biochemical parameters such as carotenoid content, chlorophyll content and nitrate reductase activity were also estimated. The plants were harvested after two months of inoculation. After termination of the experiment, plants were rinsed with normal water followed by air drying, and different growth parameters such as weight, length and yield were determined. Subsequently, the dry matter was calculated 24 h after oven drying at 60 °C.

For the assessment of egg masses, plant roots were immersed for 15 min in 0.015% Phloxine B, which specifically stains the gelatinous matrix of nematode egg masses bright red, and the egg masses per root system were counted [45].

The number of eggs/egg masses were determined by randomly selecting ten healthy and uniform sized egg masses from each root system and shaking in 1% NaOCI solution for 3 min. The egg suspension was then stained (acid fuchsin) and sieved through 200 and 500 mesh sieves (75 and 26 μm) with gentle tap water flow to collect debris on the first sieve and eggs on second [61]. The released eggs were collected in a 50 mL water suspension. The number of eggs per egg mass was counted in 1 ml using a light microscope under low power (10×).

A 250 g sample of well-mixed soil from each plant was processed by Cobb’s sieving and decanting method, followed by Baermann funnel method to determine the final nematode population in soil [62]. Nematode suspensions were collected after 24 h, and the number of nematodes was counted in five aliquots of 5 mL of suspension from each sample. The means of the five counts were used to calculate the population of nematodes per 250 g of soil.

The root-knot index was evaluated by counting the number of galls on each root system on 0-5 scale according to Taylor and Sasser [63].

### 4.5. Chlorophyll and Carotenoid Contents

The chlorophyll content in the fresh leaves was determined by the method of Mackinney [64]. One gram of fresh cut leaves of the sample was ground to a fine pulp using a mortar and pestle after adding 20 mL of 80% acetone. The obtained mixture was then centrifuged at 5000 rpm for 5 min and the supernatant was collected in a volumetric flask. The residue was washed three times using 80% acetone, and each washing was collected in the same volumetric flask. The final volume was made up to the mark using 80% acetone. Absorbance was observed at wavelengths of 645 and 663 nm for chlorophyll, and 480 and 510 nm for carotenoids against a blank (80% acetone) using a spectrophotometer (UV 1700, Shimadzu, Japan). The chlorophyll and carotenoid content present in the extract (mg g^−1^ tissue) was calculated using the following equation:

$$\mathrm{Total}\;\mathrm{chlorophyll}\;\mathrm{content}=20.2(A_{645})+8.02(A_{663})\times \left(\frac{v}{1000\times W}\right)$$

$$\mathrm{Carotenoid}\;\mathrm{content}=7.6(A_{480})-1.49(A_{510})\times \left(\frac{v}{1000\times D\times W}\right)$$

A_480,_ A_510,_ A_645,_ A_663_ = Absorbance of extract at given wavelengths (480, 510, 645, and 663 nm, respectively)

V = Final volume of the extract

W = Fresh weight of leaf sample

D = Length of path of light

### 4.6. Nitrate Reductase Activity (NRA)

The activity of nitrate reductase in fresh leaves was estimated by following the Jaworski [65] method. The leaves were chopped, and 200 mg were weighed and transferred to plastic vials. To each vial, 2.5 mL of phosphate buffer pH 7.5 and 0.5 mL of potassium nitrate solution were added followed by the addition of 2.5 mL of 5 % of isopropanol. These vials were kept in a BOD incubator for incubation for 2 h at 28 ± 2 °C in the dark. To 0.4 mL of incubated mixture in a test tube, 0.3 mL each of sulphanilamide solution and NED-HCl were added and left for 20 min for color development. The mixture was diluted to 5 mL by distilled water (DW). The absorbance was read at 540 nm using a spectrophotometer (UV 1700, Shimadzu, Japan). A blank was run simultaneously with each sample. A standard curve was plotted by using known graded concentrations of NaNO_2_ (sodium nitrite) solution. The absorbance of each sample was compared with that of the calibration curve, and NR activity (nMg^−1^ h^−1^) was calculated.

### 4.7. Estimation of Hydrogen Peroxide (H_2_O_2_)

The concentration of hydrogen peroxide was measured as per the procedure of Alexieva et al. [66]. One hundred mg leaf were squashed with 0.1 percent (w/v) trichloroacetic acid in 1.0 mL and then centrifuged at 10,000 rpm at 4 °C for 30 min. The solution was composed of 0.5 mL of supernatant, 0.1 M potassium phosphate buffer 0.5 mL and 2 mL of 1 M KI reagent. The chemical process was carried out in the dark for an hour, and the absorbance was estimated at 390 nm. The concentration of H_2_O_2_ was calculated and plotted in μmol gm^−1^ fresh weight by a standard curve.

### 4.8. Estimation of Phenol Content

Phenol content was determined by the homogenization of 2 mL 80% methanol with 100 mg leaf fragment at 70 °C and 15 min stirring [67]. Later, 200 μL of methanolic extract was mixed with 2 mL of distilled water followed by 50 μL of Folin-Ciocalteau reagent. The prepared solution was kept at 25 °C and blue coloration was detected at 725 nm using a UV-Visible spectrophotometer. Gallic acid was chosen as the standard. The phenol content was calculated as μg mg^−1^ gallic acid (GAE) equivalent.

### 4.9. Estimation of Peroxidase (POX)

Fresh leaves (100 mg) were blended in 2 mL of 0.1 M phosphate buffer (pH 7.0) at 4 °C. The prepared sample was centrifuged for 15 min at 4 °C and 16,000 g. A sample (0.5 mL) of the supernatant was added to 1.5 mL of 0.05 M pyrogallol and 0.5 mL of 1% H_2_O_2_, and the sample was incubated for enzyme activity at room temperature (28 ± 2 °C). The shift in absorbance was noted at 30 s intervals for 3 min and 420 nm, and the activity of the enzyme was noted in units gm^−1^ fresh weight [68].

### 4.10. Estimation of Superoxide Dismutase (SOD)

A 50 mM sodium phosphate buffer (pH 7.0) containing 1 m methylene diamine tetraacetic acid (EDTA) and 0.25 per cent (w/v) polyvinyl pyrrolidone (PVP) was applied to fresh leaf samples (100 mg). The prepared samples were centrifuged at 10,000 g for 15 min and 4 °C. Three ml of reaction mixture, 0.3 mL supernatant, 50 mM sodium phosphate buffer (pH 7.8), 75 μM NBT, 10 μM EDTA, 22.0 μM riboflavin and 13 mM L-methionine were incubated at 35 °C for 30 min at 4000 lx, to determine enzyme activity. The activity of SOD was spectrophotometrically evaluated at 560 nm according to the Beauchamp and Fridovich [69] procedure. SOD activity was represented in units mg^−1^ fresh weight.

### 4.11. Estimation of malondialdehyde (MDA)

The MDA content was determined by the use of Heath and Packer technique [70]. Three ml of 0.1% trichloroacetic acid (TCA) were blended with a fresh leaf sample (100 mg) and centrifuged at 10,000 g for 10 min. The centrifuged sample (300 μL) was added to 1.2 mL 2-thiobarbituric acid 0.5% (w/v) and trichloroacetic acid 20% *(w/v*). The homogenized blend was incubated at 95 °C for 30 min then centrifuged for 10 min. The collected supernatant was left for some time on a bench until it reached room temperature and the absorbance was read at 600 nm and 532 nm. The MDA content was represented in μmol gm^−1^ fresh weight.

### 4.12. Statistical Analysis

The experimental data were analyzed using SPSS17.00 (SPSS Inc., Chicago, IL, USA), statistical software via one-way analysis of variance (ANOVA). Differences within the treatments were compared by Duncan’s multiple range test for significance. According to Duncan’s multiple range test (DMRT), at *p* ≤ 0.05 means in every vertical line depicted by the same letter are not significantly different.

## Figures and Tables

**Table 1:** Impact of individual, concomitant and sequential inoculation of *Meloidogyne incognita* and *Pseudomonas fluorescens* on plant growth parameters of tomato cv. K-21 in pots.

Table	Legth (cm)			Weight (g)					
				Fresh			Dry		
	Shoot	Root	Total	Shoot	Root	Total	Shoot	Root	Total
Pf	59.2 ^a^ ± 2.60	28.3 ^a^ ± 1.45	87.5 ^a^ ± 3.13	57.4 ^a^ ± 2.30	23.8 ^a^ ± 1.30	81.2 ^a^ ± 3.92	21.6 ^a^ ± 1.00	8.2 ^a^ ± 0.55	29.8 ^a^ ± 1.23
Pf + Mi	42.0 ^d^ ± 2.10	20.0 ^d^ ± 1.12	62.0 ^d^ ± 2.56	34.6 ^d^ ± 1.76	14.9 ^d^ ± 0.80	49.5 ^d^ ± 1.79	13.0 ^d^ ± 0.66	3.7 ^d^ ± 0.39	16.7 ^d^ ± 0.87
Mi15→Pf	32.0 ^e^ ± 1.89	15.5 ^e^ ± 0.63	47.5 ^e^ ± 2.10	25.5 ^e^ ± 1.32	10.3 ^e^ ± 0.67	35.8 ^e^ ± 1.37	10.7 ^e^ ± 0.70	2.7 ^e^ ± 0.47	13.4 ^e^ ± 0.71
Pf15→Mi	47.0 ^c^ ± 2.12	23.6 ^c^ ± 1.25	71.6 ^c^ ± 2.89	42.0 ^c^ ± 1.96	18.0 ^c^ ± 0.90	60.0 ^c^ ± 3.10	16.0 ^c^ ± 0.82	5.0 ^c^ ± 0.50	21.0 ^c^ ± 0.95
UIC UUC	26.2 ^f^ ± 1.67 54.7 ^b^ ± 2.42	12.3 ^f^ ± 0.90 26.5 ^b^ ± 1.20	38.5 ^f^ ± 2.00 81.2 ^b^ ± 3.15	23.0 ^f^ ± 1.30 50.7 ^b^ ± 2.16	8.5 ^f^ ± 0.42 22.3 ^b^ ± 1.12	31.5 ^f^ ± 1.35 73.2 ^b^ ± 3.57	7.9 ^f^ ± 0.48 19.4 ^b^ ± 0.96	1.4 ^f^ ± 0.57 7.0 ^b^ ± 0.51	9.3 ^f^ ± 0.53 26.4 ^b^ ± 1.20

Each value is the mean of five replicates; Means in each column followed by the same letter are not significantly different according to Duncan’s Multiple Range Test (DMRT) at *p* ≤ 0.05. → Indicates the sequence of inoculation of *M. incognita* (Mi)/*P. fluorescens* (Pf) 15 days prior. + Indicates simultaneous inoculation. UUC: Untreated Uninoculated Control. UIC: Untreated inoculated Control.

**Table 2:** Impact of individual, concomitant and sequential inoculation of *Meloidogyne incognita* and *Pseudomonas fluorescens* on yield and biochemical parameters of tomato cv. K-21 in pots.

Treatment	Pollen Fertility (%)	Yield/Plant (g)	Chlorophyll Content (mg/g)	Carotenoid Content (mg/g)	NRA (nMg^−1^ h^−1^)
Pf	91.6 ^a^ ± 3.00	385 ^a^ ± 11.42	2.95 ^a^ ± 0.046	0.924 ^a^ ± 0.007	332 ^a^ ± 9.22
Pf + Mi	72.0 ^d^ ± 2.62	250 ^d^ ± 8.65	2.12 ^d^ ± 0.048	0.689 ^d^ ± 0.011	250 ^d^ ± 6.89
Mi15→Pf	61.0 ^e^ ± 2.50	194 ^e^ ± 4.22	1.69 ^e^ ± 0.058	0.540 ^e^ ± 0.012	220 ^e^ ± 6.12
Pf15→Mi	79.4 ^c^ ± 2.75	300 ^c^ ± 9.00	2.32 ^c^ ± 0.043	0.816 ^c^ ± 0.009	285 ^c^ ± 7.50
UIC UUC	47.0 ^f^ ± 1.75 89.5 ^b^ ± 2.89	148 ^f^ ± 3.78 355 ^b^ ± 9.76	1.12 ^f^ ± 0.072 2.78 ^b^ ± 0.039	0.272 ^f^ ± 0.019 0.880 ^b^ ± 0.012	142 ^f^ ± 3.25 307 ^b^ ± 8.54

Each value is the mean of four replicates; Means in each column followed by same letter are not significantly different according to Duncan’s Multiple Range Test (DMRT) at *p* ≤ 0.05. NRA: Nitrate reductase activity. → indicates the sequence of inoculation of *M. incognita* (Mi)/*P. fluorescens* (Pf) 15 days prior. + indicates simultaneous inoculation; UIC: Untreated inoculated Control. UUC: Untreated Uninoculated Control.

**Table 3:** Impact of individual, concomitant and sequential inoculation of Meloidogyne *incognita* and *Pseudomonas fluorescens* on nematode multiplication in tomato cv. K-21 in pots.

Treatment	Eggmasses/Root	Eggs/Eggmass	Nematodepopulation/250 g Soil	Root-Knot Index
Pf	0 ^e^ ± 0.00	0 ^e^ ± 0.00	0 ^e^ ± 0.00	0 ^e^ ± 0.00
Pf + Mi	94 ^c^ ± 2.45	160 ^c^ ± 2.50	790 ^c^ ± 19.20	2.0 ^c^ ± 0.13
Mi15→Pf	118 ^b^ ± 3.20	189 ^b^ ± 2.85	907 ^b^ ± 22.42	2.4 ^b^ ± 0.11
Pf15→Mi	64 ^d^ ± 1.89	118 ^d^ ± 3.82	640 ^d^ ± 17.50	1.4 ^d^ ± 0.15
UIC UUC	175 ^a^ ± 3.80 0 ^e^ ± 0.00	266 ^a^ ± 12.34 0 ^e^ ± 0.00	1608 ^a^ ± 32.34 0 ^e^ ± 00	5.0 ^a^ ± 0.57 0 ^e^ ± 0.00

Each value is the mean of five replicates. Means in each column followed by the same letter are not significantly different according to Duncan’s Multiple Range Test (DMRT) at *p* ≤ 0.05. → indicates the sequence of inoculation of *M. incognita* (Mi)/ *P. fluorescens* (Pf) 15 days prior. + indicates simultaneous inoculation. UIC: Untreated inoculated Control. UUC: Untreated Uninoculated Control.

## Data Availability

The data presented in this study are available within the article.
